# 

**Published:** 1890-03

**Authors:** 


### Bristol Medico-Chirurgical Journal.

### MARCH, 1890.

### CONTENTS.

ORIGINAL PAPERS: *Page*

Personal Methods employed in the Radical Cure of Hernia—Inguinal, Femoral and Umbilical. By J. GREIG SMITH ... 1

The Sequelæ of Suppurative Otitis Media. By F. H. EDGEWORTH, B.A., M.B. (Cantab), B.Sc. (Lond.) ... 18

CLINICAL RECORDS:

A Case of Factitious Urticaria. By HENRY WALDO, M.D. Aber., M.R.C.P. Lond. ... 29

Factitious Urticaria. By ALFRED J. HARRISON, M.B. (Lond.) ... 32

REVIEWS OF BOOKS ... 36

NOTES ON PREPARATIONS FOR THE SICK ... 46

THE INTERNATIONAL MEDICAL CONGRESS ... 49

PERISCOPE ... 54

SCRAPS ... 71

